# Spontaneous Tumor Lysis Syndrome: A Case Report and Critical Evaluation of Current Diagnostic Criteria and Optimal Treatment Regimens

**DOI:** 10.1177/2324709615603199

**Published:** 2015-08-25

**Authors:** Alicia C. Weeks, Michelle E. Kimple

**Affiliations:** 1 William S. Middleton Memorial Veterans Hospital, Madison, WI, USA; 2 University of Wisconsin, Madison, WI, USA

**Keywords:** spontaneous tumor lysis syndrome, allopurinol, rasburicase, uric acid, renal failure

## Abstract

Tumor lysis syndrome (TLS) is a known complication of malignancy and its treatment. The incidence varies on malignancy type, but is most common with hematologic neoplasms during cytotoxic treatment. Spontaneous TLS is thought to be rare. This case study is of a 62-year-old female admitted with multisystem organ failure, with subsequent diagnosis of aggressive B cell lymphoma. On admission, laboratory abnormalities included renal failure, elevated uric acid (20.7 mg/dL), and 3+ amorphous urates on urinalysis. Oliguric renal failure persisted despite aggressive hydration and diuretic use, requiring initiation of hemodialysis prior to chemotherapy. Antihyperuricemic therapy and hemodialysis were used to resolve hyperuricemia. However, due to multisystem organ dysfunction syndrome with extremely poor prognosis, the patient ultimately expired in the setting of a terminal ventilator wean. Although our patient did not meet current TLS criteria, she required hemodialysis due to uric acid nephropathy, a complication of TLS. This poses the clinical question of whether adequate diagnostic criteria exist for spontaneous TLS and if the lack of currently accepted guidelines has resulted in the underestimation of its incidence. Allopurinol and rasburicase are commonly used for prevention and treatment of TLS. Although both drugs decrease uric acid levels, allopurinol mechanistically prevents formation of the substrate rasburicase acts to solubilize. These drugs were administered together in our patient, although no established guidelines recommend combined use. This raises the clinical question of whether combined therapy is truly beneficial or, conversely, detrimental to patient outcomes.

## Background

### Diagnostic Criteria for Tumor Lysis Syndrome (TLS) and Spontaneous TLS

TLS is an oncologic emergency resulting from massive tumor lysis that produces an array of metabolic abnormalities, which may include hyperkalemia, hyperphosphatemia, hypocalcemia, and hyperuricemia. Although most often seen with hematologic malignancies in the context of cytotoxic therapy, it has also been shown to occur in a multitude of other neoplasm types with significant tumor burden and robust sensitivity to traditional chemotherapy. Rare, but noteworthy case reports illustrate that TLS can occur in alternative settings, such as following use of relatively weak tumoricidal agents such as glucocorticoids, administration of monoclonal therapy independently of traditional cytotoxic agents, and with radiation treatment.^1-3^ Similar to cases involving hematologic malignancies treated with conventional cytotoxic therapy, these reports demonstrate the propensity for TLS in the context of widespread disease and a vigorous therapeutic response. Spontaneous TLS, or TLS in the absence of cytotoxic therapy, has also been reported, but it is considered much less common. Hyperphosphatemia is less commonly seen with spontaneous TLS, as the phosphate released in the spontaneous setting is thought to be reused in the generation of new neoplastic cells that would be less likely to assimilate with chemotherapy administration.^4^ As hypocalcemia results from serum calcium binding excess phosphorus, it is reasonable to presume this metabolic derangement would occur less frequently with spontaneous TLS as well. Thus, the true incidence of each TLS type remains difficult to quantify.^5^

Although first observed in 1963, standardized objective definitions of TLS were not evident until the early 1990s, with Hande and Garrow quantifying risk factors and classifying laboratory (LTLS) versus clinical (CTLS) manifestations.^6^ The most widely accepted current definition of TLS proposed by Cairo and Bishop is more elaborate, with allowances for pretreatment electrolyte abnormalities (Table 1), additional criteria for diagnosis within 7 days of treatment, and a proposed grading system to quantify the severity of the syndrome.^7^

**Table 1.** Cairo–Bishop Definition of Tumor Lysis Syndrome.

Laboratory tumor lysis syndrome (LTLS)—Must contain 2 or more of the following criteria^a^	
Uric acid	≥8.0 mg/dL or 25% increase from baseline
Potassium	≥6.0 mmol/L or 25% increase from baseline
Phosphorus	≥4.5 mg/dL or 25% increase from baseline
Calcium	≤7.0 mg/dL or 25% decrease from baseline
Clinical tumor lysis syndrome (CTLS)—Meets criteria for LTLS + one of the following	
Creatinine	≥1.5 times the upper limit of normal
Cardiac arrhythmia/sudden death	
New-onset seizure(s)	

a Within 3 days before or 7 days after cytotoxic therapy.

More recently, Montesinos et al proposed that serum creatinine elevation be incorporated into the LTLS criteria, with oliguria and dialysis more appropriately placed in the CTLS category.^8^ However, the Montesinos proposals were made with respect to acute myeloid leukemia patients, and it is unclear how widely these criteria are applied outside of this limited context. In contrast to the more inclusive diagnostic criteria proposed by Montesinos and colleagues, Howard and colleagues endorsed more restrictive standards with respect to the laboratory definition.^5^ These included both reverting back to the pre–Cairo-Bishop era in recognizing only absolute abnormalities in metabolic derangements rather than relative increases from the patient’s baseline, and that the 2 or more abnormalities required for LTLS must be observed within a strict 24-hour period. On the other hand, Howard and colleagues supported expanding the clinical criteria to include any symptomatic hypocalcemia.

### Treatment Regimens

Uric acid is a natural product of purine catabolism. As humans lack the enzyme urate oxidase, uric acid is also a final oxidation product, which is primarily renally excreted. Massive tumor lysis, whether spontaneous or in the context of cytotoxic therapy, can result in profound hyperuricemia, thus placing patients at risk for renal failure secondary to uric acid nephropathy. Although traditionally defined as an obstructive nephropathy due to intrarenal uric acid crystal deposition, uric acid is now also believed to cause renal injury via endothelial dysfunction and inflammation.^9^ Prior to the era of rasburicase—a recombinant form of urate oxidase—aggressive intravenous fluid hydration and allopurinol were the mainstay of both prevention and treatment of hyperuricemic complications in adults. Alkalization of urine was also once used to increase the solubility of uric acid, but due to concurrent precipitation of calcium phosphate, it is no longer recommended as routine treatment. Although only approved for adult use in the United States recently, rasburicase has been used off-label to prevent and treat hyperuricemia-associated complications in TLS for some time. However, its role in the setting of allopurinol therapy continues to evolve and remains a subject of some debate.

**Figure 1.**
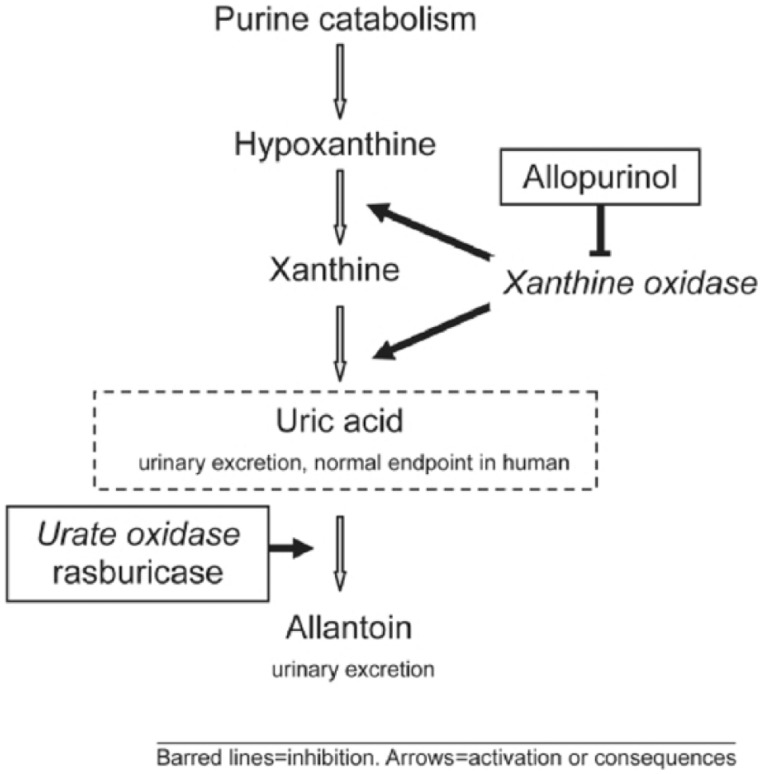
Purine catabolism and mechanisms of action of allopurinol and rasburicase. Reprinted from Pession et al.^11^

Although both allopurinol and rasburicase function as hypouricemic agents, their mechanisms of action are distinct (Figure 1). Allopurinol inhibits the enzyme xanthine oxidase, preventing new formation of uric acid, but it is ineffective against preformed compound. As a result, it is not able to lower plasma uric acid (PUA) levels rapidly. Furthermore, allopurinol use results in accumulation of hypoxanthine and xanthine, the latter of which is less soluble than uric acid and may also cause renal failure due to obstructive nephropathy.^10^

Rasburicase degrades uric acid into allantoin, a compound 5 to 10 times more soluble than uric acid. As rasburicase uses uric acid as its substrate, it is able to lower PUA levels much more rapidly than allopurinol. Manufacturer guidelines for rasburicase state that use with allopurinol is not indicated and expert review advises against concurrent administration of these drugs based on their mechanisms of action.^9^ Despite these recommendations, these agents are often used in combination in clinical practice, which is suspected to occur with the intention to minimize the amount of rasburicase needed, thus reducing cost. No studies have compared allopurinol versus rasburicase in spontaneous TLS, presumably due to the spontaneous nature of the condition. Data on the optimal treatment of spontaneous TLS remains limited to case reports.^12,13^ A recent review article discusses a number of studies exploring the appropriate treatment of adults at high risk for TLS for which rasburicase has been deemed standard of care even without a full understanding of the cost versus benefit.^14^ Only 2 of these studies incorporated combination therapy, with one employing only a stepwise regimen involving both drugs.^15,16^

Cortes et al performed a phase III randomized study comparing use of (*a*) rasburicase (RSB) alone, (*b*) rasburicase (days 1-3) followed by allopurinol (days 3-7) (RSB + ALLP), and (*c*) allopurinol (ALLP) alone.^15^ Table 2 summarizes both the time to PUA control and the PUA response rates found in this study. The only statistically significant difference in PUA response rates was between the rasburicase and allopurinol monotherapy regimens.

**Table 2.** Time to PUA Control and Response Rates for Cortes et al Treatment Regimens^a^.

	Treatment Regimen		
	RSB	RSB + ALLP	ALLP
Time to PUA control^b^	4 hours	4 hours	27 hours
PUA response rate^c^	87%	78%	66%
95% CI	80% to 94%	70% to 87%	56% to 76%

Abbreviations: PUA, plasma uric acid; RSB, rasburicase; ALLP, allopurinol; CI, confidence interval.

a Data from Cortes et al.^15^

b Time from initial medication administration to PUA level ≤7.5 mg/dL.

c Defined as PUA level ≤7.5 mg/dL for all measurements from days 3 to 7, no unplanned extension of antihyperuricemic treatment, or missing samples (more than 2 consecutive samples or day 7 sample).

The incidence of LTLS in this phase III study was significantly lower with rasburicase monotherapy in comparison to allopurinol monotherapy, but there were inadequate numbers of patients with CTLS to draw statistically significant conclusions of the effect on clinical outcomes. Interestingly, no statistically significant difference was found in the PUA response rate between stepwise therapy and allopurinol monotherapy, although the author of this study attributed this finding to both missing data and the need for treatment extension beyond 5 days rather than a lack of PUA control. The statistical significance of differences between rasburicase monotherapy and stepwise combination therapy with respect to PUA response rate and incidence of LTLS were not reported.

Eaddy et al performed a retrospective study comparing economic outcomes of concurrent combination allopurinol–rasburicase therapy to rasburicase monotherapy by quantifying both overall length of stay (LOS), critical care LOS, and hospitalization costs.^16^ As shown in Table 3, an overall hospitalization cost comparison was equivocal between the 2 regimens, although a trend toward higher cost was seen with combination therapy. LOS with combination therapy was significantly longer than with monotherapy.

**Table 3.** Length of Stay (LOS) and Costs for Rasburicase Monotherapy Versus Combined Therapy With Allopurinol^a^.

	Rasburicase Monotherapy	Combination Therapy^b^	*P* Value
Hospitalization cost	$35 843	$46 672	.0059
Mean duration of rasburicase use (days)	2.7	2.1	.0820
Mean LOS (days)	10	15.4	.0067
Mean critical care LOS (days)	2.4	2.9	.3389

a Data from Eaddy et al.^16^

b Allopurinol and rasburicase.

## Our Experience: Case Report

A 62-year-old white female presented with a several week history of general malaise, intermittent febrile episodes for several months, and development of both anorexia and acute metabolic encephalopathy 48 to 72 hours prior to admission. A mammogram study performed earlier that month cited ominous appearing masses of the breast and axilla, with recommendations for biopsy and flow cytometry. Physical exam was significant for encephalopathy, tachycardia, jaundice, and palpable masses in the axilla. Acute renal failure, lactic acidosis, hyperuricemia, and leukocytosis with numerous immature forms were noted on admission (Table 4). Chest x-ray was without infiltrates, and a noncontrast computed tomography of the abdomen/pelvis showed gallbladder wall thickening.

**Table 4.** Serum Laboratory Data^a^.

	Reference Range	72 Hours before Admission	On Admission
Sodium (mmol/L)	135-145	140	133
Chloride (mmol/L)	98-108	103	93
Carbon dioxide (mmol/L)	22-33	24	16
Blood urea nitrogen (mg/dL)	6-24	10	50
Creatinine (mg/dL)	0.6-1.1	1.54	7.09
Calcium (mg/dL)	8.4-10.5	10.5	10.7
Albumin (g/L)	3.2-4.4	—	2.4
Uric acid (mg/dL)	2.6-7.2	—	20.7
Ionized calcium (mmol/L)	1.09-1.30	—	1.29
Estimated GFR (mL/min/1.73 m^2^)	≥60	34	6
White blood cells (thouands/µL)	4.0-10.5	17.7	29.9
Bands (%)	<10	24	12
Neutrophils (%)	45-82	37	38
Monocytes (%)	4-13	9	20
Hemoglobin (g/dL)	12.1-15.8	10.4	9.1
Hematocrit (%)	35.8-46.5	29.6	27.2
Platelets (thousands/µL)	154-393	131	108
Aspartate aminotransferase (U/L)	0-45	—	227
Alkaline phosphatase (U/L)	0-133	—	450
Alanine aminotransferase (U/L)	0-60	—	81
Total bilirubin (mg/dL)	0.2-1.2	—	4.1
Direct/indirect bilirubin (mg/dL)	0-0.2/0-1.1	—	2.0/2/1
Lipase (IU/L)	0-36	—	45
International normalized ratio	0.8-1.2	1.5	1.6
Protime (seconds)	9.8-13.4	16.7	18
Myoglobin	0-200	—	493

a Potassium, glucose, phosphorus, ionized calcium, lymphocyte %, eosinophil %, basophil %, troponin, CKMB, and BNP were all within normal limits.

The patient was admitted to the intensive care unit and treated with broad-spectrum antibiotics and high-flow intravenous fluids (IVF). Oliguric renal failure persisted despite IVF and robust IV diuretics, necessitating hemodialysis within 72 hours. Urinalysis showed amorphous urates, which in combination with profound hyperuricemia and oliguric renal failure was consistent with uric acid nephropathy. Right axillary node core biopsy, bone marrow biopsy, and cerebrospinal fluid analyses confirmed diagnosis of diffuse aggressive large B cell non-Hodgkin’s lymphoma with central nervous system involvement.

One dose of rasburicase, 6 mg IV, was ordered concurrent with allopurinol (100 mg IV given daily for 5 days) following the initial hemodialysis session. Chemotherapy was initiated on day 4 with systemic cyclophosphamide, adriamycin, vincristine, and prednisone as well as intrathecal methotrexate and hydrocortisone. Rituximab therapy was started on day 7. Impending respiratory failure necessitated endotracheal intubation and the patient remained ventilator dependent thereafter.

Increasing oxygen requirement prompted a bronchoscopy, revealing pulmonary hemorrhage syndrome and raised concern of a concurrent infectious etiology. Despite a modified antibiotic regimen, the patient’s condition continued to deteriorate. Ultimately, her family elected for palliative measures only, and the patient expired.

## Discussion

### Combination Treatment Regimens

The effect of clinical outcomes in adult patients receiving combination rasburicase–allopurinol therapy was limited primarily to a single study that focused on economic and health utilization outcomes.^16^ This study did not find the combination regimen advantageous from a cost perspective and actually revealed it was detrimental with respect to LOS. Rasburicase is recommended for monotherapy use based on its contradictory mechanism of action to allopurinol. As rasburicase use is typically limited to either the treatment of existing TLS or patients at high risk of developing TLS, it would also seem reasonable to minimize other potential renal impairments such as xanthine nephropathy, thus making concurrent allopurinol therapy ill-advised. Further safety and efficacy studies are needed to assess combined allopurinol and rasburicase use, but no current evidence supports combination therapy and detrimental effects of such therapy remain a clinical concern.

### Diagnostic Criteria for TLS and Spontaneous TLS

The patient was admitted with renal failure secondary to uric acid nephropathy in the context of undiagnosed diffuse large B cell lymphoma. However, despite a uric acid of over 20 mg/dL and resultant renal failure necessitating hemodialysis prior to cytotoxic therapy, she did not meet the currently accepted diagnostic criteria for TLS.

We believe that not only are the currently accepted criteria insufficient to accurately diagnose spontaneous TLS but also underestimate its incidence. This underestimation of spontaneous TLS is best illustrated by findings of Hsu et al in 2004.^17^ This retrospective review of 926 patients with acute renal failure based the diagnosis of spontaneous TLS on the presence of uric acid nephropathy, elevated lactate dehydrogenase, and biopsy proven malignancy, rather than simply focusing on metabolic derangements. Spontaneous TLS was found in 1.08% of these patients, making the incidence uncommon, but certainly not as extraordinary as the medical literature implies.^18^

Therefore, it is proposed that in the absence of cytotoxic therapy, that patients with hyperuricemia (UA ≥ 8.0 mg/dL) in the presence of suspected malignancy with elevated lactate dehydrogenase (>2 × ULN), acute oliguric or anuric renal failure despite adequate volume resuscitation without evidence of postobstructive cause, and urinary uric acid to creatinine ratio greater than 1.0 be considered spontaneous TLS until proven otherwise. In simplistic terms, if a patient is suspected to have an undiagnosed malignancy with evidence of significant tumor breakdown that is causing end organ damage, empiric treatment should be initiated to minimize that damage while the diagnosis is clarified. Although definitions and criteria are useful in many respects, understanding the pathophysiology associated with massive tumor lysis and applying this knowledge to a patient’s clinical presentation is far more valuable than any classification system or predictive algorithm.

It is reasonable to assume that patients with spontaneous TLS may not have a malignancy diagnosis on presentation, as seen with our patient. As it is also not unusual for patients with organ dysfunction of unclear etiology to be admitted to internists, simplification of criteria could assist generalists in minimizing and possibly preventing permanent renal damage. As the morbidity, mortality, and cost associated with renal replacement therapy is substantial, the impact of earlier diagnosis could be significant on many levels.^19,20^ In many respects, this proposal is not markedly different from that of administering steroids for suspected temporal arteritis nodosa prior to biopsy-proven disease or providing empiric antibiotic therapy when bacterial meningitis is suspected, but cerebrospinal fluid analysis is pending.
